# Transforming work or eroding social capital? How reliance on artificial intelligence drives workplace involution

**DOI:** 10.3389/fpsyg.2026.1842907

**Published:** 2026-08-14

**Authors:** Ruochen Huang

**Affiliations:** School of Business, Fuzhou Polytechnic University, Fuzhou, China

**Keywords:** anxiety, artificial intelligence reliance, employee psychology, involution, performance expectations, social capital, social comparison, workplace behavior

## Abstract

The rapid integration of artificial intelligence (AI) into organizational contexts is reshaping how employees work, interact, and respond to changing workplace dynamics. Although AI is often expected to improve efficiency and reduce workload, emerging evidence points to a potential dark side, as AI adoption may intensify competitive pressure within organizations. Motivated by this paradox, this study examines how AI reliance may erode workplace social capital by fostering involution—an excessive and inefficient form of competition characterized by escalating effort and diminishing returns. This research focuses on digitally enabled and highly competitive workplace settings, where employee performance and outputs are more visible, transparent, and comparable. We propose that, from employees’ perspective, AI reliance is associated with higher levels of involution through elevated performance expectations and anxiety. Using survey data from employees in China, the study hypotheses were tested using structural equation modeling. The results support the proposed model and indicate a significant sequential mediation pattern linking AI reliance, performance expectations, employee anxiety, and workplace involution. Notably, the direct relationship between AI reliance and involution is not significant, suggesting that AI reliance is associated with employees’ defensive competitive behavior primarily through employees’ perception of external evaluative pressures and internal psychological responses. These findings highlight the unintended social consequences of AI adoption in organizations, and underscore the importance of protecting employee wellbeing, communicating realistic performance expectations, and maintaining workplace social capital when implementing AI in digitally enabled organizations.

## 1 Introduction

Generative artificial intelligence (AI) is increasingly integrated into workplace practices, reshaping work structures, collaboration, and interpersonal dynamics (Pereira et al., 2023). Early academic discourse optimistically suggested that AI would liberate human labor; improve employee wellbeing (Jarrahi, 2018; Jia et al., 2024); and enhance employees’ structural, relational, and cognitive social capital (Deng et al., 2022). However, contemporary workplaces are witnessing an emerging paradox. Although the use of AI at work has reportedly doubled since 2023 to support organizational growth (Niederhoffer et al., 2025), growing AI reliance may paradoxically increase competitive pressures. This phenomenon echoes the Jevons Paradox in economic theory, which suggests that technological improvements in efficiency often lead to increased rather than decreased resource consumption (Luccioni et al., 2025). Similarly, in AI-enabled workplaces, efficiency gains may not reduce work intensity but instead lead to escalating efforts.

Under such conditions, workplace involution emerges as a state of hyper-competition characterized by excessive and self-defeating effort within saturated evaluative systems (Yi et al., 2022). Such dynamics may gradually erode workplace social capital, which is typically conceptualized as the norms and networks that support collective action (Woolcock, 2001). In organizational contexts, workplace social capital refers to the relational resources embedded in employee ties—such as trust, shared norms, and network ties—that enable coordination and cooperation among employees. As involution intensifies, employees may rely less on mutual cooperation and more on efforts to maintain their relative standing, shifting organizational attention from relationship-building to intensified individual competition (Salin et al., 2022), thereby undermining the relational foundations of workplace social capital (Leana and Van Buren, 1999). Against this backdrop, an important question emerges: Why does not labor-saving technology truly save us? More specifically, through what psychological and behavioral mechanisms might AI integration fuel such irrational competition dynamics?

Despite the rapidly growing literature on AI in the workplace (Pereira et al., 2023; Jia et al., 2025; Lin et al., 2025), prior research has largely focused on AI’s instrumental benefits (Tong et al., 2021; Jia et al., 2024) or its direct psychological costs for employees (Koo et al., 2021; Ban et al., 2024). Although some studies have begun to examine the risks of AI reliance more directly, such as cognitive offloading (Gerlich, 2025) and reduced cooperation (Klingbeil et al., 2024), comparatively little attention has been paid to how AI reliance may be associated with changes in perceived performance-related pressures and competitive dynamics. Moreover, although prior studies suggest that elevated performance expectations may induce employee anxiety (Xi et al., 2021; Smollan and Mooney, 2024), and status threat may further amplify dysfunctional coworker behavior (Reh et al., 2018), existing research rarely integrates these factors into a unified employee-level mechanism explaining how AI reliance may translate into excessive and inefficient competition.

To address these gaps, the present study examines workplace involution as a potential unintended consequence of AI reliance. Specifically, focusing on digitally intensive and highly competitive workplace contexts, we propose that AI reliance is associated with higher perceived performance expectations, which are further related to increased employee anxiety and ultimately to experienced workplace involution. By testing this sequential mechanism, this study reveals a cognitive–emotional pathway linking AI reliance to workplace involution, and advances the understanding of how labor-saving technologies may paradoxically intensify competitive pressures rather than alleviate them.

## 2 Theoretical framework and hypothesis development

### 2.1 Adoption and reliance on AI in the workplace

AI is increasingly embedded in workplace practices, with the aim of improving task execution and performance (Pereira et al., 2023). Early applications positioned AI primarily as an auxiliary tool that automated routine tasks, enabling employees to focus on more complex and creative work (Zirar, 2023). However, as AI has been integrated into performance-evaluation systems (Sardi and Modarelli, 2026), task-allocation processes (Mishra and Varshney, 2024), and project management (Taboada et al., 2023), it has become increasingly central to everyday work, with employees relying on it more extensively to organize tasks, support decisions, and coordinate collaboration (Eckhardt et al., 2025).

Baier (1986) defined reliance as a continued relationship on the basis of one party’s dependable habits toward the other. In the context of emerging technologies, scholars have extended this concept to describe individuals’ dependence on technological systems. For example, AI reliance has been conceptualized as the extent to which individuals consistently depend on AI systems to perform tasks reliably and over time (Elippatta and Intezari, 2025), as well as the likelihood that human decision-makers follow AI-generated recommendations (Guo et al., 2025). Similarly, Eckhardt et al. (2025) suggest that reliance can be inferred when users consistently follow AI advice. In this study, we conceptualize AI reliance in the workplace as employees’ habitual dependence on AI systems to perform both routine and non-routine tasks.

As employees increasingly rely on AI-enabled tools in their daily work, they may complete tasks more quickly and, in many contexts, produce higher-quality outputs (Przegalinska et al., 2024). As AI use becomes more widespread, such gains may extend beyond traditionally high-performing employees and become more common across the workforce (Noy and Zhang, 2023). In digitalized workplaces, these improvements are also more visible, and employees are more likely to perceive their work processes and outputs as easily observable and comparable by others (Grisold et al., 2024). Meanwhile, AI is often associated with rapid processing, immediate feedback, and enhanced decision quality, which may make delays and errors more salient and less tolerable in digitalized workplaces (Li et al., 2025; Jarrahi, 2018). Therefore, high performance may increasingly be perceived as a baseline expectation rather than an exceptional outcome. In turn, this perception may lead employees to infer that leaders are recalibrating acceptable performance standards, resulting in higher perceived performance expectations.

In addition, technostress research suggests that AI-related uncertainty, continuous technological change, and perceived skill-substitution risks can trigger anxiety regarding career development and job security (Bondanini et al., 2020). As employees increasingly rely on AI systems to perform tasks and evaluate performance, concerns about keeping pace with technological change may heighten feelings of tension and apprehension. Accordingly, this research predicts:

*H1a*: AI reliance is positively related to performance expectations.

*H1b*: AI reliance is positively related to employee anxiety.

### 2.2 Performance expectations and workplace anxiety

Prior research has highlighted the motivational benefits of high performance expectations—such as those concerning enhanced self-efficacy and improved performance (Carmeli and Schaubroeck, 2007). However, do high performance expectations always lead to high performance outcomes? Emerging evidence highlights their potential downsides.

Drawing on cognitive appraisal theory (Lazarus and Folkman, 1984), individuals evaluate work demands as either challenges or threats by assessing whether these demands are relevant to their wellbeing and whether sufficient resources are available to cope with them. When such expectations are perceived as exceeding available resources, they may evoke anxiety—an affective state characterized by feelings of tension and worry in response to uncertain or potentially threatening situations (Cisler et al., 2010; Spielberger, 2019). In workplace contexts, this form of anxiety typically manifests as a negative emotional state related to work demands and concerns about successfully completing job tasks (Zhang et al., 2022). These dynamics may be intensified in AI-enabled work environments, where transparency and monitoring make employees more aware of evaluative standards and heighten their sense of being continuously assessed (Kellogg et al., 2020), encouraging upward social comparison and intensifying competitive pressure. Under such conditions, rather than functioning as motivational signals, leaders’ high performance expectations may be perceived as chronic stressors, thereby amplifying employee anxiety and defensive competitive behaviors (Xi et al., 2021; Smollan and Mooney, 2024). Thus, we propose that:

*H2*: Performance expectations are positively related to employee anxiety.

### 2.3 Involution in the workplace

Originating in the Chinese context, the concept of involution (*nei juan*, literally meaning “coiling inward”) describes a pattern of excessive and inefficient competition in which individuals continuously escalate effort without achieving meaningful improvements in relative standing, leading to diminishing returns on personal investment (Dou et al., 2022; Li, 2021).

In the era of AI, workplace involution increasingly manifests as a zero-sum competitive dynamic (Chen and Zhang, 2022). Employees intensify their efforts not primarily to create additional value but rather to avoid falling behind peers or becoming technologically obsolete (Zhang and Liao, 2025). Heightened performance expectations further amplify these concerns. When perceived performance expectations continue to escalate, employees may feel that maintaining their relative standing requires greater effort, even when such effort does not generate proportional performance gains. From this perspective, escalating performance pressure may make employees more likely to show a self-defensive competitive tendency, thereby increasing their perceived involvement in workplace involution (Mitchell et al., 2019).

According to social comparison theory (Festinger, 1954), individuals rely on others’ performance as a reference for evaluating their own behavior under conditions of uncertainty. Anxiety heightens attention to peers’ actions, motivating employees to adjust their effort in response to others rather than to objective task demands (Reh et al., 2018). As employees face technology-related uncertainty and anxiety, imitating the behaviors of high-performing or majority peers may become a psychologically safe coping strategy (Laland, 2004). Under such conditions, employees may show a greater tendency toward defensive competition and become more likely to be involved in workplace involution. These dynamics may trigger a collective escalation process commonly described as the “theater effect.” Similar to a situation in which individuals seated in the front rows stand up to gain a better view, prompting those behind them to stand as well (Yu, 2018), employees may continuously adjust their effort in response to others’ behavior, such that one individual’s anxiety-driven overinvestment in effort creates pressure for others to respond similarly. Over time, this self-reinforcing cycle may draw employees into excessive competition with limited productivity gains, thereby reinforcing workplace involution.

Taken together, these arguments suggest that:

*H3a*: Performance expectations are positively related to workplace involution.

*H3b*: Employee anxiety is positively related to workplace involution.

*H4*: Performance expectations and employee anxiety sequentially mediate the relationship between AI reliance and workplace involution.

## 3 Methods

### 3.1 Participants and procedures

Data were collected through an online survey administered via Credamo,^1^ a crowdsourcing platform widely used for behavioral and psychological research (e.g., Xu et al., 2023; Huang and Lin, 2024). Participants were recruited from Credamo’s “Data Market” in late 2025.

The study complied with standard ethical guidelines for voluntary participation and data confidentiality. Before completing the questionnaire, respondents were informed about the purpose of the study, the confidentiality of their responses, and the voluntary nature of participation. Participants received a small monetary reward upon completion. Inadequate responses to embedded attention-check questions were automatically excluded by the platform. After screening, the final sample consisted of 550 valid responses.

Among the respondents, 38.00% were male and 62.00% were female. Most participants were aged between 21 and 40 years (86.00%). In terms of monthly income, 19.64% earned 3,000–5,000 RMB, 43.82% earned 5,000–10,000 RMB, and 22.73% earned 10,000–20,000 RMB. The majority of respondents held a bachelor’s degree or higher (84.55%).

### 3.2 Measures

All measures were adapted from established scales. The original English items were translated into Chinese using Brislin’s (1986) translation and back-translation procedure, and minor discrepancies were resolved through team discussion. A pre-survey with 50 participants was conducted to ensure the clarity and readability of the questionnaire. Feedback indicated that the items were easy to understand and posed no response barriers. All constructs were measured on a 5-point Likert scale, and only the formal survey data were used for the main analyses.

#### 3.2.1 Core constructs

##### 3.2.1.1 AI reliance

AI reliance was measured using items adapted from the 5-point Likert scale developed by Hou et al. (2025), which assesses individuals’ degree of reliance on generative AI during problem-solving and collaboration. The original six-item scale was adapted to the workplace context. A sample item is: “When performing my work tasks, I spend a significant amount of time interacting with AI to obtain necessary information” (Cronbach’s *α* = 0.758).

##### 3.2.1.2 Performance expectation

Performance expectations were measured using the three-item scale developed by Podsakoff et al. (1990), which captures employees’ assessment of the extent to which their leaders expect them to achieve high-quality and superior performance (Cronbach’s *α* = 0.68). A sample item is: “I think my leader has high performance expectations of me.” The general reliability criterion for scales is Cronbach’s *α* > 0.7. Given that the Performance Expectation scale has only three items and Cronbach’s alpha is highly sensitive to scale length (Tavakol and Dennick, 2011), 0.6 was deemed the acceptable lower bound (Hair et al., 2019).

##### 3.2.1.3 Anxiety

Employee anxiety was measured using the 13-item scale developed by McCarthy et al. (2016), which assesses anxiety related to work pressure (Cronbach’s *α* = 0.948). A sample item is: “I am overwhelmed by thoughts of doing poorly at work.”

##### 3.2.1.4 Involution

Workplace involution (Cronbach’s *α* = 0.863) was measured using the 13-item Involution Perception Scale developed by Zhang et al. (2024). A sample item is: “Most people around me continue to put in additional effort even though they already met the minimum job requirements.”

#### 3.2.2 Control variables

Following prior research on organizational psychology and workplace behavior (Judge et al., 2001; Xi et al., 2021), in addition to demographic variables (gender, age, education level, and income), we included job satisfaction, job control, and organizational commitment as control variables, as these factors are closely associated with employees’ affective responses and behavioral outcomes (Riketta, 2002; Du et al., 2018). Specifically, prior research suggests that job satisfaction is associated with work-related anxiety (Matud et al., 2024) and employees’ extra-role, collective-oriented workplace behaviors (Organ and Ryan, 1995). Organizational commitment may affect employees’ stress-coping strategies (Meyer and Maltin, 2010) and job performance (Riketta, 2002), while job control influences how employees feel and behave when confronting high demands (van der Doef and Maes, 1999).

Job satisfaction was measured using the six-item scale developed by Tsui et al. (1992); a sample item is: “Are you satisfied with your job?” Job control was assessed using the six-item scale from Gonzalez-Mulé and Cockburn (2017); a sample item is: “My job allows me to make many decisions on my own.” Finally, organizational commitment was measured using the five-item scale developed by Gao-Urhahn et al. (2016); a sample item is: “I feel proud to be a member of this organization.” All items were rated on a 5-point Likert scale, and reliability coefficients were satisfactory (Cronbach’s *α* = 0.904, 0.912, and 0.937 for job satisfaction, job control, and organizational commitment, respectively).

#### 3.2.3 Scale purification and item parceling

Item purification was conducted to enhance the reliability, validity, and parsimony of the measures, while preserving adequate coverage of the underlying constructs (Wieland et al., 2017). Following prior recommendations, 0.50 was used as a minimum reference threshold for standardized factor loadings. Decisions on item retention also considered construct representativeness, item redundancy, and overall model fit (Brown, 2015; Hair et al., 2019). Based on this process, two items from the AI reliance scale and four items from the workplace involution scale were excluded. The final measurement model included the following indicators: AI reliance (4 items), performance expectations (3 items), anxiety (13 items), and workplace involution (9 items).

Given the relatively large number of items for anxiety and workplace involution scale, item parceling was applied using the balanced method (Wu and Wen, 2011). Specifically, the 13 anxiety items were combined into four parcels, while the nine involution items were combined into three parcels. Items were first ranked by factor loadings and then assigned to parcels using a high-to-low alternating procedure to ensure balanced loadings and variances across parcels.

### 3.3 Data analysis method

To test the hypotheses, the analyses proceeded in four steps: preliminary analyses, measurement model assessment, structural model testing, and mediation analysis.

Descriptive statistics and Pearson’s correlations were first conducted in jamovi (version 2.6.45.0). Internal consistency reliability was assessed using Cronbach’s alpha coefficients, while composite reliability (CR) and average variance extracted (AVE) were calculated to further evaluate construct reliability and convergent validity. Confirmatory factor analysis (CFA) was then used to evaluate the construct validity of the measurement model. Model fit was assessed using *χ*^2^/df, the comparative fit index (CFI), Tucker–Lewis index (TLI), standardized root mean square residual (SRMR), and root mean square error of approximation (RMSEA), with acceptable fit indicated by *χ*^2^/df < 5, CFI and TLI ≥ 0.90, RMSEA ≤ 0.08, and SRMR ≤ 0.08 (Bentler, 1990; Steiger, 1990). Potential common method bias was examined using Harman’s single-factor test and the unmeasured latent method construct (ULMC) approach. Structural equation modeling was subsequently conducted in R using the lavaan package, with control variables included, and indirect effects were tested via bootstrapping with 5,000 resamples. Mediation was considered significant when the 95% confidence interval (CI) did not include zero.

## 4 Results

### 4.1 Descriptive statistics and correlations

Table 1 presents the descriptive statistics and Pearson correlations among the study variables. AI reliance was positively correlated with performance expectations (*r* = 0.242, *p* < 0.001), anxiety (*r* = 0.352, *p* < 0.001), and workplace involution (*r* = 0.368, *p* < 0.001). Overall, the correlations among the primary variables were significant in the expected directions, providing preliminary support for the proposed relationships. In addition, variance inflation factor (VIF) values were examined to assess multicollinearity. All VIF values were below 10, indicating that multicollinearity was not a serious concern (Chatterjee et al., 2006).

**Table 1:** Means, standard deviations, and correlations among study variables.

Variable	Mean	SD	1	2	3	4	5	6	7	8	9	10	11
AI reliance	3.633	0.637	(0.758)										
Performance expectations	4.097	0.604	0.242 ***	(0.68)									
Anxiety	3.205	0.973	0.352***	0.171***	(0.948)								
Involution	3.587	0.646	0.368***	0.434***	0.582***	(0.863)							
Job satisfaction	3.467	0.876	−0.086*	0.046	−0.556***	−0.303***	(0.904)						
Job control	3.429	0.942	−0.100*	0.034	−0.535***	−0.249***	0.782***	(0.912)					
Organization commitment	3.545	1.027	−0.073	0.083	−0.526***	−0.231***	0.878***	0.815***	(0.937)				
Gender	1.62	0.486	0.081	0.006	0.055	0.061	−0.017	0.020	−0.013				
Age	2.55	0.835	0.033	0.075	−0.202***	−0.060	0.201***	0.240***	0.229***	−0.063			
Income	2.91	0.985	−0.034	0.197***	−0.204***	0.000	0.207***	0.193***	0.178***	−0.125**	0.387***		
Education level	3.99	0.687	−0.010	0.017	−0.043	−0.004	−0.018	−0.046	−0.077	0.072	−0.068	0.308***	

*N* = 550. *p < 0.05, **p < 0.01, ***p < 0.001. Gender: 1 = male; 2 = female. Age: 1 = 18–20 years old; 2 = 21–30 years old; 3 = 31–40 years old; 4 = 41–50 years old; 5 = ≥51 years old. Income: 1 = <3,000 yuan; 2 = 3,000–5,000 yuan; 3 = 5,000–10,000 yuan; 4 = 10,000–20,000 yuan; 5 = > 20,000 yuan. Education Level: 1 = Middle school or below; 2 = High school; 3 = Associate degree; 4 = Bachelor’s degree; 5 = Master’s degree or above. Internal consistency reliabilities are on the diagonal, in parentheses.

### 4.2 Measurement model

Confirmatory factor analysis was conducted to evaluate the measurement model. To assess discriminant validity, we compared the hypothesized four-factor model with a series of alternative measurement models. As shown in Table 2, the hypothesized four-factor model showed a good fit to the data (*χ*^2^ = 206.783, df = 71, *χ*^2^/df = 2.91, CFI = 0.972, TLI = 0.964, RMSEA = 0.059, SRMR = 0.045) and fit the data substantially better than all alternative factor models, thereby supporting the discriminant validity of the key variables. In addition, as shown in Table 3, all CR values exceeded 0.70, and all AVE values were above 0.50 except for performance expectations (0.464). Given that CR values above 0.70 are considered acceptable and AVE values between 0.36 and 0.50 may still indicate acceptable convergent validity (Fornell and Larcker, 1981), these results provide further support for construct reliability and convergent validity of the core constructs.

**Table 3:** Reliability and convergent validity tests for the core constructs.

Variable	Factor loading	*α*	AVE	CR
AI reliance	0.539–0.831	0.758	0.500	0.744
Performance expectations	0.669–0.697	0.680	0.464	0.722
Anxiety	0.606–0.837	0.948	0.581	0.947
Involution	0.638–0.823	0.863	0.511	0.904

Factor loadings are standardized. *α*, Cronbach’s alpha; AVE, average variance extracted; CR, composite reliability.

**Table 2:** Comparison of measurement models.

Model	Factor	*χ* ^2^	df	*χ*^2^/df	RMSEA	SRMR	CFI	TLI
Baseline model	AR, PE, AN, IN	206.783	71	2.912	0.059	0.045	0.972	0.964
ULMC model	AR, PE, AN, IN, common method factor	191.533	70	2.736	0.056	0.046	0.975	0.967
Three-factor model	AR, PE + AN, IN	578.343	74	7.815	0.111	0.115	0.894	0.870
Three-factor model	AR, PE, AN+IN	1369.941	74	18.513	0.178	0.143	0.729	0.667
Three-factor model	AR + PE, AN, IN	557.705	74	7.537	0.109	0.105	0.899	0.876
Two-factor model	AR + PE, AN+IN	1615.334	76	21.254	0.192	0.159	0.678	0.614
Two-factor model	AR + PE + AN, IN	1022.155	76	13.449	0.150	0.151	0.802	0.763
One-factor model	AR + PE + AN+IN	2047.440	77	26.590	0.216	0.174	0.588	0.513

AR, AI reliance; PE, performance expectations; AN, anxiety; IN, involution. “+” indicates that factors were combined in the alternative measurement models.

Given that all variables were measured using self-reported data, common method bias may be a potential concern. We first conducted Harman’s single-factor test, and the results showed that the first factor accounted for 33.02% of the total variance, which is below the suggested threshold of 40% (Podsakoff et al., 2003). However, given the limited sensitivity of this test (Zhou and Long, 2004), we further employed a ULMC approach following prior research (Chen et al., 2017). Specifically, a common method factor was added to the measurement model with equal indicator loadings and orthogonality to the substantive constructs (Tang and Wen, 2020). The ULMC model (*χ*^2^ = 191.533, df = 70, CFI = 0.975, RMSEA = 0.056) was compared with the baseline CFA model to examine whether model fit improved substantially. The results showed that the inclusion of the method factor led to only a minimal improvement in model fit (ΔCFI = 0.003; ΔRMSEA = 0.003), indicating that common method bias is unlikely to materially affect the results (Richardson et al., 2009).

### 4.3 Structural model and hypothesis testing

**Figure 1:**
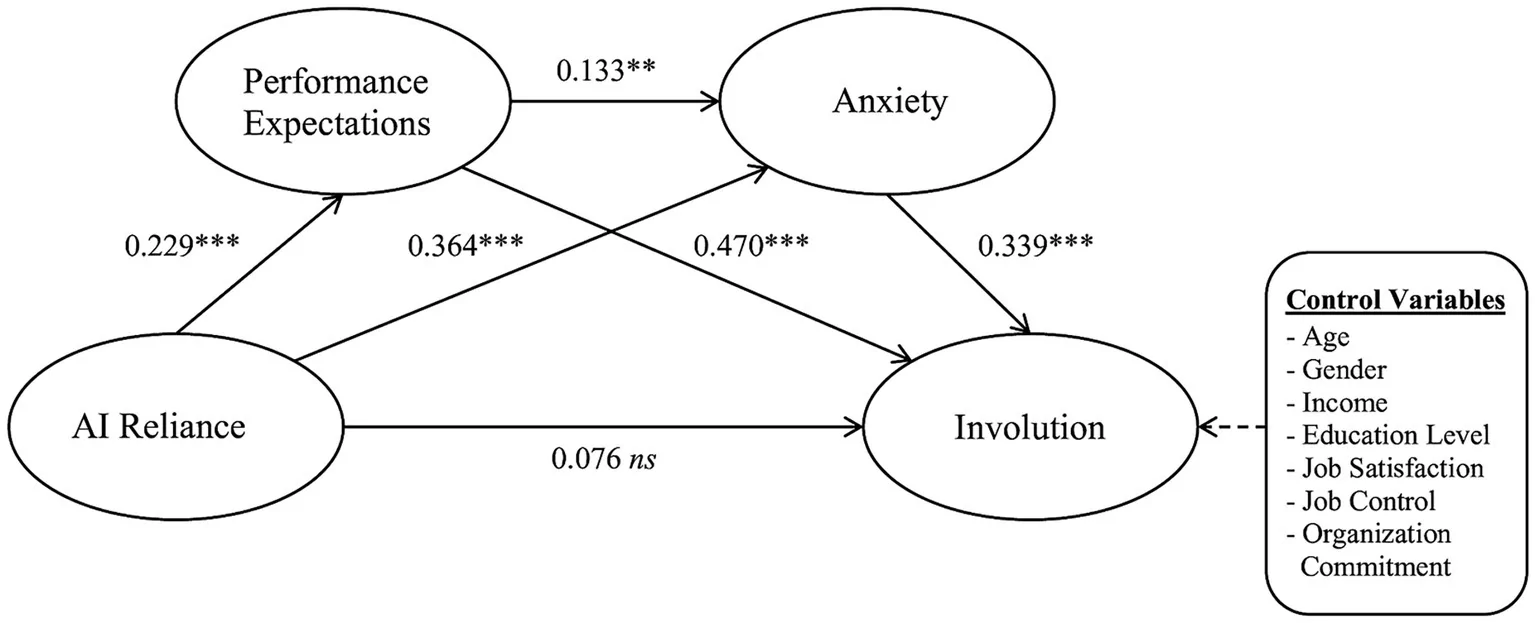
Path coefficients of the hypothesized model. *N* = 550. Standardized path coefficients are reported. ***p < 0.001, **p < 0.01, *p < 0.05. ns, non-significant. The dashed arrow indicates the combined effect of control variables.

Structural equation modeling was conducted to test the hypothesized relationships, and the model demonstrated satisfactory fit to the data (*χ*^2^ = 1208.79, df = 529, *χ*^2^/df = 2.29, CFI = 0.949, TLI = 0.944, RMSEA = 0.048, SRMR = 0.044). The standardized path coefficients are illustrated in Figure 1. AI reliance showed significant positive associations with performance expectations (*β* = 0.229, *p* < 0.001) and anxiety (*β* = 0.364, *p* < 0.001), supporting H1a and H1b. Performance expectations were positively related to anxiety (*β* = 0.133, *p* < 0.01) and involution (*β* = 0.470, *p* < 0.001), providing support for H2 and H3a, respectively. Anxiety was also significantly related to involution (*β* = 0.339, *p* < 0.001), supporting H3b. However, the direct path from AI reliance to involution was not significant (*β* = 0.076, *p* > 0.05), suggesting that this relationship may be better explained through indirect pathways.

### 4.4 Mediation analysis

Indirect effects were tested using a bias-corrected bootstrap procedure with 5,000 resamples. The results (Table 4) revealed a significant sequential mediation effect whereby AI reliance indirectly influenced involution through performance expectations and anxiety (AI reliance → performance expectations → anxiety → involution; *β* = 0.017, 95% CI [0.006, 0.028]). Therefore, H4 was supported.

**Table 4:** Bootstrapped direct and indirect effects.

Effect	Path	*β*	95% CI
Direct effect	AR → IN	0.0832	[−0.028, 0.187]
Sequential mediation	AR → PE → AN→IN	0.0169	[0.006, 0.028]
Indirect effect	AR → PE → IN	0.1231	[0.053, 0.193]
Indirect effect	AR → AN→IN	0.0896	[0.047, 0.132]

*β* refers to standardized effect estimates for the direct and indirect effects. Confidence intervals were obtained using a bias-corrected bootstrap procedure with 5,000 resamples. AR, AI reliance; PE, performance expectations; AN, anxiety; IN, involution; CI, confidence interval.

In addition, two simple mediation pathways were observed. The indirect effect of AI reliance on involution through performance expectations was significant (AI reliance → performance expectations → involution; *β* = 0.123, 95% CI [0.053, 0.193]), as was the indirect effect through anxiety (AI reliance → anxiety → involution; *β* = 0.090, 95% CI [0.047, 0.132]). In all cases, the confidence intervals did not include zero. The direct effect of AI reliance on involution was not significant (*β* = 0.083, 95% CI [−0.028, 0.187]), indicating that the association between AI reliance and involution was mainly explained by the indirect pathways tested in the present model. Taken together, the results supported our hypotheses.

## 5 Discussion

Using the original survey data, the present study examined how employees’ AI reliance is associated with experienced workplace involution in digitally enabled and highly competitive workplaces. Consistent with our predictions, the results supported a significant sequential mediation pathway linking AI reliance, performance expectations, anxiety, and workplace involution. Within this framework, the two significant simple indirect effects further unpack the cognitive and emotional aspects of this process: the indirect path through performance expectations highlights how AI reliance may shape employees’ cognitive appraisal of heightened performance demands, whereas the indirect path through anxiety underscores the emotional response associated with such pressure. Together, these findings suggest that AI reliance is associated with workplace involution primarily through a cognitive–emotional mechanism rather than a direct behavioral pathway.

The present research offers several theoretical contributions. First, it extends the emerging literature on AI and workplace involution. Existing AI-at-work research has focused predominantly on augmentation, productivity, and performance gains (Noy and Zhang, 2023; Przegalinska et al., 2024), whereas the broader involution literature has focused more on conceptualization, measurement (Dou et al., 2022), and downstream outcomes than on its technology-related antecedents in workplace settings (Yin et al., 2023). The limited work that has begun to consider the link between AI and workplace involution has remained at the level of public discourse and text-mining analysis (Huo and Siau, 2025). By examining workplace involution as employees’ experience of excessive and inefficient competition, the present study extends this literature beyond the instrumental consequences of AI use to the level of competitive perceptions and behavioral tendencies at work. Second, prior research has separately linked AI-related work contexts to employee anxiety (Huang and Gursoy, 2024), performance expectations to employee wellbeing (Smollan and Mooney, 2024), and status-related concerns to dysfunctional coworker behavior (Reh et al., 2018). The present study advances this literature by integrating these previously disconnected relationships into a sequential cognitive–emotional mechanism. This finding advances a process-based explanation, in which involvement in involution may also be understood as a defensive behavioral response to technology-related uncertainty and intensified evaluative pressure. Finally, the non-significant direct effect between AI reliance and involution is theoretically informative, as it suggests that its effects depend on how rising performance demands are interpreted and psychologically processed by employees.

The findings also offer valuable practical insights. First, these findings highlight the importance of considering employee wellbeing when implementing AI technologies. Although AI systems may improve organizational efficiency, their long-term value also depends on whether they benefit employees in a sustainable way (Bughin and Manyika, 2019). Organizations and managers may therefore need to set realistic and development-oriented performance benchmarks, provide adequate technological and psychological support, and avoid translating AI-enabled productivity gains into escalating performance expectations. Second, as social exchange theory suggests, when employees perceive organizational care and support, they may develop a stronger sense of reciprocal obligation and trust, which may in turn encourage more constructive attitudes and behaviors (Cropanzano and Mitchell, 2005). Therefore, a supportive work environment marked by trust, knowledge sharing, and interpersonal support may help employees navigate technological uncertainty and adapt more effectively to digitalized work settings. Finally, as work becomes increasingly technology-mediated, employees are also required to cope with its associated cognitive and emotional demands (Bondanini et al., 2020). Therefore, at the employee level, self-regulation strategies may help employees regulate emotional responses to technological uncertainty, continuously update their digital skills, and adapt more effectively to AI-enabled work.

Despite these contributions, several limitations should be acknowledged. First, the cross-sectional design of this research limits our ability to draw causal inferences about the relationships among the variables. Future research could adopt longitudinal or experimental designs to examine how AI reliance influences employee perceptions and behaviors over time. Second, limitations also arise from the study’s measurement approach and sample scope. Although we conducted additional tests to assess common method bias, reliance on self-reported measures may still raise concerns about same-source bias. In addition, respondents recruited from online platforms may be relatively more digitally literate, which could shape how they perceive and rely on AI at work. Moreover, this study focuses on employees’ self-reported AI reliance and their experienced workplace involution, rather than directly examining organizational-level AI reliance or macro-level involution dynamics. Future research could therefore incorporate multi-source and multilevel data, as well as objective behavioral indicators (e.g., organizational records or digital trace data) to examine whether individual perceptions of AI reliance and involution may aggregate into broader team or organizational-level dynamics. Finally, the present study does not identify the boundary conditions under which the proposed mechanism may be stronger or weaker. The observed associations may not apply equally across all AI use contexts, organizational settings, or industries. Future research could examine moderating factors such as employee resilience, organizational culture, and relational resources, and further explore whether the proposed mechanism varies across industries or cultural contexts.

## 6 Conclusion

In conclusion, this study provides empirical evidence that employees’ AI reliance may be associated with their experienced workplace involution through a cognitive–emotional mechanism involving perceived performance expectations and anxiety. Rather than directly generating involution, AI reliance may influence employees’ appraisal of performance demands, thereby triggering psychological responses that subsequently influence workplace behavior. By uncovering the psychological processes underlying this relationship, the research advances understanding of how digital technology may reshape competitive dynamics in contemporary workplaces, and highlights the importance of balancing technological efficiency with employee wellbeing and sustainable, cooperative workplace environment in the age of AI.

## Data Availability

The raw data supporting the conclusions of this article will be made available by the authors, without undue reservation.

## Footnotes

1
https://www.credamo.com
